# Elevated AA/EPA Ratio Represents an Inflammatory Biomarker in Tumor Tissue of Metastatic Colorectal Cancer Patients

**DOI:** 10.3390/ijms20082050

**Published:** 2019-04-25

**Authors:** Valeria Tutino, Valentina De Nunzio, Maria Gabriella Caruso, Nicola Veronese, Dionigi Lorusso, Marta Di Masi, Maria Lucrezia Benedetto, Maria Notarnicola

**Affiliations:** 1 Laboratory of Nutritional Biochemistry, National Institute of Gastroenterology “S. de Bellis” Research Hospital, Castellana Grotte, 70013 Bari, Italy; valeria.tutino@irccsdebellis.it (V.T.); valentinadx@hotmail.it (V.D.N.); m.benedetto85@gmail.com (M.L.B.); 2 Ambulatory of Clinical Nutrition, National Institute of Gastroenterology “S. de Bellis” Research Hospital, Castellana Grotte, 70013 Bari, Italy; gabriella.caruso@irccsdebellis.it (M.G.C.); ilmannato@gmail.com (N.V.); 3 Surgery Unit, National Institute of Gastroenterology “S. de Bellis” Research Hospital, Castellana Grotte, 70013 Bari, Italy; dionigi.lorusso@irccsdebellis.it; 4 Scientific Direction, National Institute of Gastroenterology “S. de Bellis” Research Hospital, Castellana Grotte, 70013 Bari, Italy; marta.dimasi@irccsdebellis.it

**Keywords:** Arachidonic acid, eicosapentaenoic acid, inflammatory biomarker, colorectal cancer, metastasis

## Abstract

Chronic inflammation increases the risk of developing certain types of cancer, such as colorectal cancer (CRC). The oxidative metabolism of polyunsaturated fatty acids (PUFAs) has a strong effect on colonic tumorigenesis and the levels of arachidonic acid (AA) and eicosapentaenoic acid (EPA) can contribute to the development of an inflammatory microenvironment. Aim of this study was to evaluate the possible differences in the AA/EPA ratio tissue levels between CRC patients with and without synchronous metastases. Moreover, the expression of the most important inflammatory enzymes and mediators, linked with the AA/EPA ratio, have been also assessed. Sixty-eight patients with CRC were enrolled in the study, of which 33 patients with synchronous metastasis. Fatty acid profile analysis in tissue samples was done to examine the levels of AA and EPA. High levels of the AA/EPA ratio were detected in tumor tissue of patients with metastatic CRC. Moreover, an increase of expression of the main enzymes and mediators involved in inflammation was also detected in the same samples. The lipidomic approach of inflammation allows to evaluate lipid homeostasis changes that occur in cancer and in its metastatic process, in order to identify new biomarkers to be introduced into clinical practice.

## 1. Introduction

Chronic inflammation creates a microenvironment suitable to the development of several complex diseases, such as cancer [1,2,3]. The release of inflammatory cells and mediators increases cancer cell survival promoting tumor progression, invasion and metastasis, but it is also able to stop the pro-apoptotic processes. Several studies have shown the relationship between colorectal cancer (CRC) and pro-inflammatory stimuli, such as cytokines, chemokines and eicosanoids, that represent the most important lipid signaling molecules involved in the regulation of inflammation and colon cancer progression [1,3,4]. Eicosanoids including prostaglandins, thromboxanes and leukotrienes, are synthesized by both omega-3 and omega-6 polyunsaturated fatty acids (PUFAs) through different enzymes, such as cyclooxygenases (COXs), lipoxygenases (LOXs) and cytochrome P450 (CYP) [5]. Eicosanoids derived from omega-3 and omega-6 exert an opposite action. It is known that eicosanoids derived from arachidonic acid (AA) have a proinflammatory action, while those derived from eicosapentaenoic acid (EPA) exert an anti-inflammatory action [6]. Therefore, an altered fatty acids profile, characterized by an excess of omega-6, creates a pro-inflammatory environment leading the cells to be more sensitive to neoplastic transformation [6,7]. 

The study of fatty acids profile in cell membrane can be considered a method to identify biomarkers associated with cancer progression [6,8,9]. Different studies have demonstrated that fatty acids play an essential role in cellular functions, such as survival, proliferation and cell death, and all these cellular processes are strongly correlated with the transformation, progression and metastasis [6,10]. Therefore, lipidomics represents an emerging approach for the characterization of cancer, allowing to evaluate the biochemical alterations happening in cell membranes [6,11]. Previously, we showed high levels of the omega-6/omega-3 ratio in colorectal tissue of metastatic CRC patients, and these differences in PUFA expression, were more marked in tumor tissue compared to surrounding normal mucosa, suggesting the important role of omega-6 in the weakening of cell membranes in metastatic tissues [12]. 

Since AA and EPA are the precursors of the most important inflammatory mediators, AA/EPA ratio is an index to better evaluate inflammation and nutritional status of cell membrane [13,14,15,16]. Several mechanisms can explain the potential pro-inflammatory effects mediated by AA/EPA ratio, in carcinogenesis and tumor proliferation. EPA seems to play an important role in suppressing inflammatory responses, competing with AA. Moreover, EPA is considered the precursor of eicosanoid mediators at lower grade of inflammation respect those derived from AA [17]. 

The oxidative metabolism of AA and EPA is tightly linked to the actions of COXs and LOXs, in colon tumorigenesis [5]. The study of the expression of COX-2, an inducible enzyme localized in sites of inflammation, as well as the study of 15-LOX-1, precursor of lipoxins with anti-inflammatory action, represent a strategy to evaluate the physiological balance of eicosanoids within the cell. COX-2 is the main enzyme that metabolizes AA, leading to the formation of prostaglandins, such as prostaglandin E2 (PGE2), and thromboxanes of the series 4 (TX-4) implicated in colon cancer. COX-2 has been also demonstrated to induce progression and metastasis in CRC affecting the matrix metalloproteinase expression [18]. Conversely, 15-LOX-1 exerts antioxidant and antimetastatic effects through the inhibition of interleukin-1β (IL-1β), tumor necrosis factor α (TNF-α) and the activation of peroxisome proliferator-activated receptor gamma (PPAR-γ) [19,20]. 

COXs and LOXs are also able to inactivate the endocannabinoid system (ECS) composed of specific cannabinoid receptors, such as CB1 and CB2, the endogenous ligands that include anandamide (AEA) and 2-arachidonoylglycerol (2-AG), and the enzymes responsible for their synthesis and degradation. Intracellular levels of AEA and 2-AG, considered the main anti-inflammatory mediators derived from AA, are regulated by enzymatic activity of COXs and LOXs [21]. Several studies have shown that inactivation of ECS, mediated by oxidation and subtraction of the endogenous ligands and by the down-regulation of the CB2 receptor (CB2-R), leads to an increase in inflammation in the CRC [22,23].

The present study was undertaken to evaluate the possible differences in the AA/EPA ratio tissue levels between CRC patients with and without synchronous metastases. Furthermore, the aim of this study was also to assess the expression of the most important inflammatory enzymes and mediators, linked with the AA/EPA ratio, having a role in CRC progression.

## 2. Results

The clinical and histopathological features of CRC patients are shown in Table 1. Sixty-eight consecutive patients (27 females and 41 males) were enrolled in the study and of these, 33 patients had synchronous metastases at the first diagnosis evaluated using computed tomography (CT).

**Figure 1:**
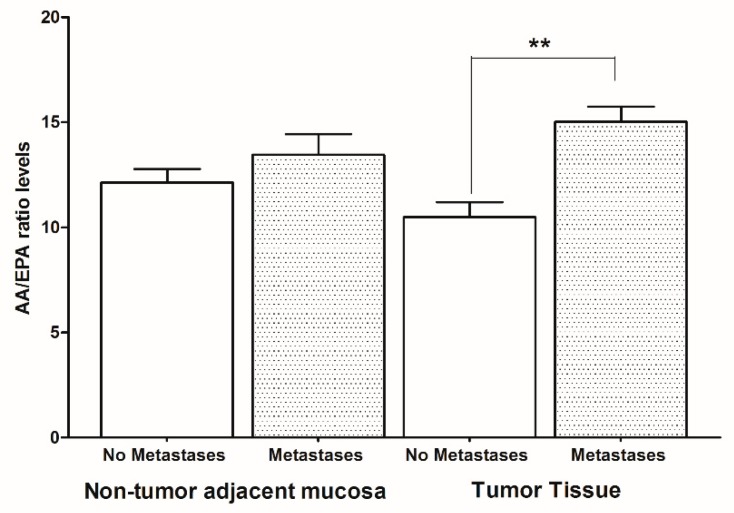
Arachidonic acid/eicosapentaenoic acid (AA/EPA) ratio levels in tumor tissue and non-tumor adjacent mucosa from colorectal cancer patients with and without metastases. Data are expressed as mean percentage ± SD. ** *p* < 0.02 (Analysis of variance (ANOVA and Tukey’s multiple comparison test).

Figure 1 shows the AA/EPA ratio levels detected using the gas chromatography method. CRC patients with metastases showed higher levels of the AA/EPA ratio compared to CRC patients without metastases, both in non-tumor adjacent mucosa and in tumor tissue. However, significant differences in the AA/EPA ratio were found only in tumor tissue, suggesting that the pro-inflammatory stimulus induces cancer cells to proliferate and metastasize. Moreover, a significant association between AA/EPA ratio levels and primary tumor stage was also detected using chi-squared test. Whereas, no association was detected between AA/EPA ratio levels and histological tumor grade.

**Figure 2:**
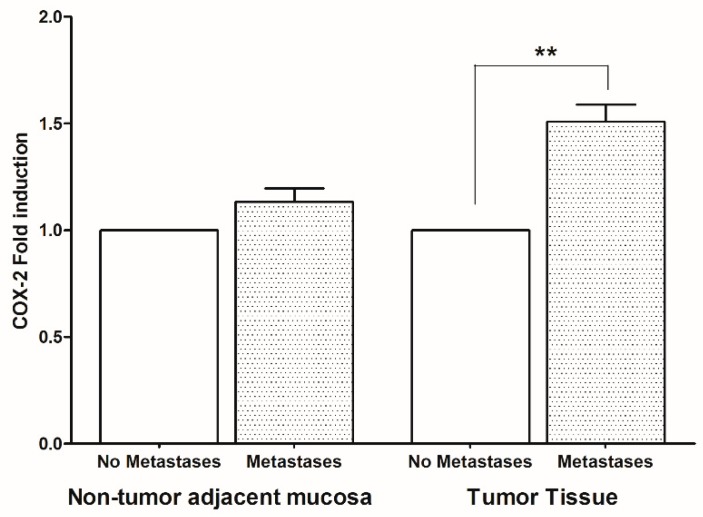
Cyclooxygenase-2 (COX-2) gene expression levels (mean ± SD) in non-tumor adjacent mucosa and tumor tissue from colorectal cancer patients with and without metastases. Data are expressed as Fold induction. ** *p* < 0.02 (Student’s *t*-test).

To evaluate whether the increase of AA/EPA ratio can be considered an inflammatory biomarker linked to the formation of metastases in CRC, the expression levels of the most important inflammatory enzymes and mediators derived from AA and EPA, were evaluated. Figure 2 shows high levels of COX-2 gene expression in patients with metastases compared to patients without metastases and these differences were statistically significant in tumor tissue. 

**Figure 3:**
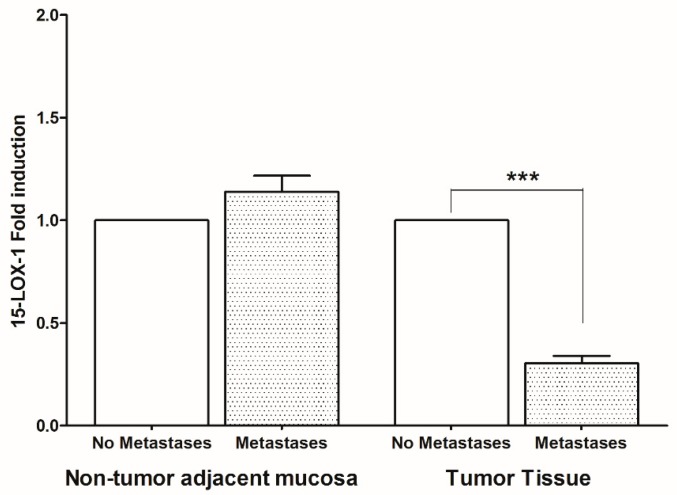
15-lipoxygenase-1 (15-LOX-1) gene expression levels (mean ± SD) in non-tumor adjacent mucosa and tumor tissue from colorectal cancer patients with and without metastases. Data are expressed as Fold induction. *** *p* < 0.005 (Student’s *t*-test).

On the contrary, in the tumor tissue of patients with metastases, a statistically significant reduction of 15-LOX-1 was found (Figure 3). 

**Figure 4:**
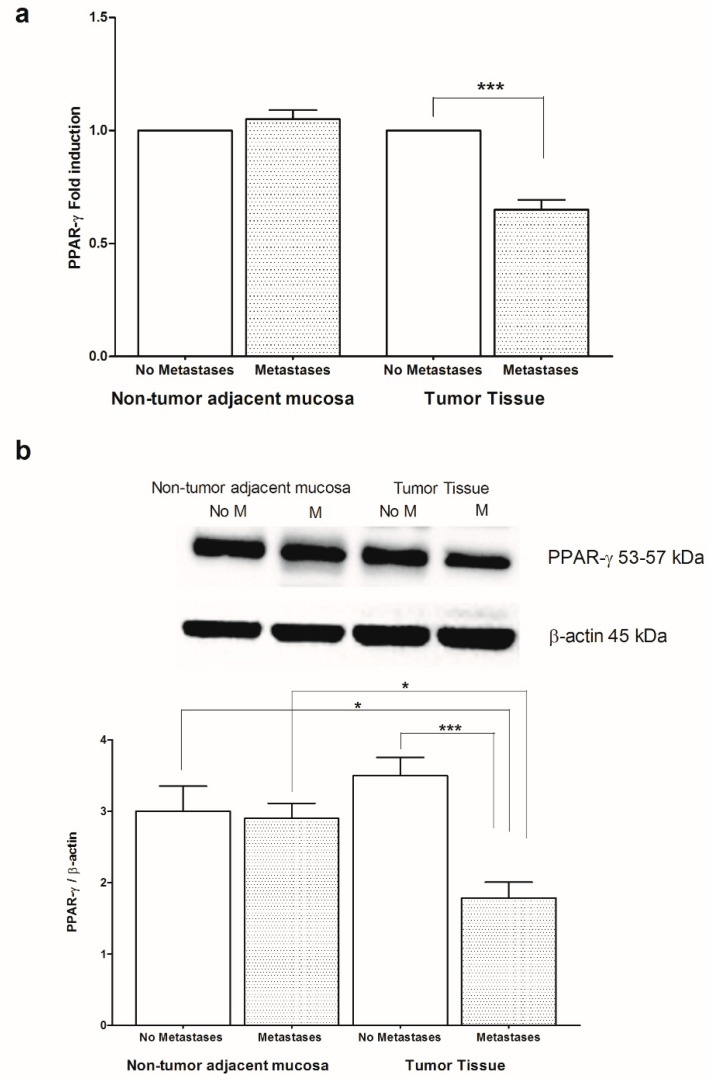
(**a**) Peroxisome proliferator-activated receptor gamma (PPAR-γ) mRNA levels in non-tumor adjacent mucosa and tumor tissue from colorectal cancer patients with and without metastases. Data are expressed as Fold induction. *** *p* < 0.005 (Student’s *t*-test). (**b**) Western blotting analysis of PPAR-γ protein in the same patients. Representative blots are also shown (No M = No Metastases; M = Metastases). * *p* < 0.05, *** *p* < 0.005 (ANOVA and Tukey’s multiple comparison test).

**Figure 5:**
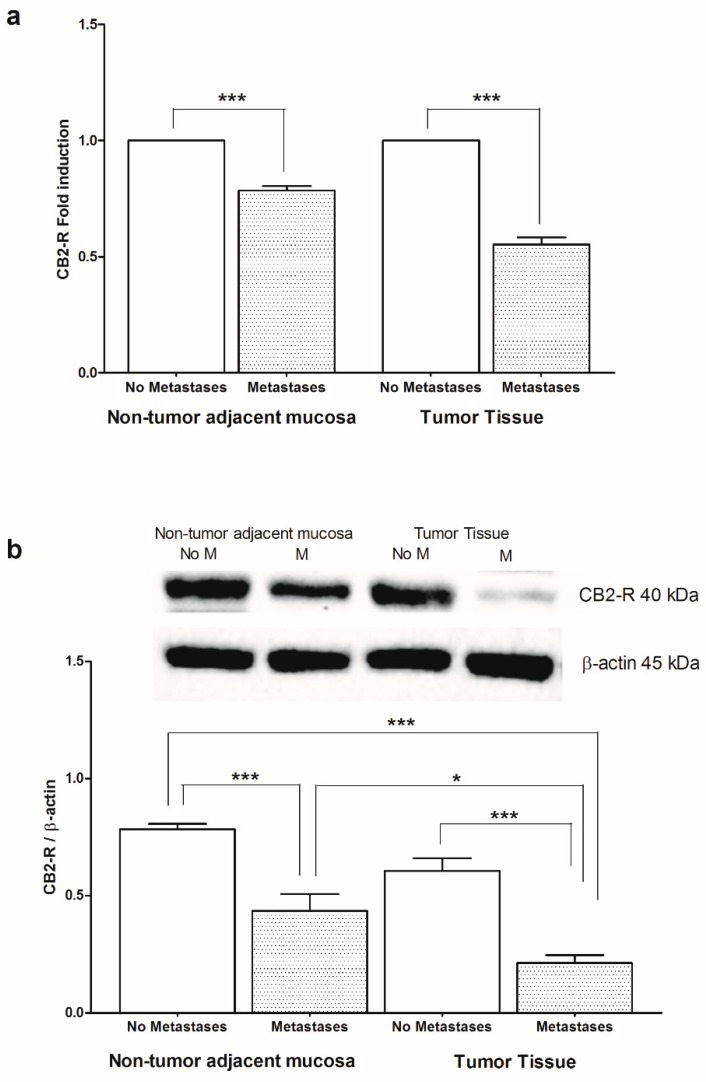
(**a**) Cannabinoid receptor 2 (CB2-R) mRNA levels in non-tumor adjacent mucosa and tumor tissue from colorectal cancer patients with and without metastases. Data are expressed as Fold induction. *** *p* < 0.005 (Student’s *t*-test). (**b**) Western blotting analysis of CB2-R protein in the same patients. Representative blots are also shown (No M = No Metastases; M = Metastases). * *p* < 0.05, *** *p* < 0.005 (ANOVA and Tukey’s multiple comparison test).

Given the anti-inflammatory role of PPAR-γ and CB2-R in colon cancer and their involvement with AA and EPA function, we wanted to evaluate the levels of mRNA and protein of PPAR-γ and CB2-R in CRC patients with and without metastases, both in non-tumor adjacent mucosa and in tumor tissue (Figure 4 and Figure 5, respectively). A statistically significant reduction, both in PPAR-γ gene and protein levels, was found in the tumor tissue of subjects with metastasis compared to those without metastases, while no differences in gene and protein expression were found in the non-tumor adjacent mucosa (Figure 4a,b). Moreover, the tumor tissue of patients with metastasis showed lower levels of PPAR-γ protein respect to corresponding “normal” adjacent mucosa (Figure 4b). 

Figure 5 shows the CB2-R gene (a) and protein (b) expression in our samples, demonstrating a statistically significant difference in CB2-R levels between patients with and without metastases both in non-tumor adjacent mucosa and in tumor tissue. In “normal” adjacent mucosa, the CB2-R protein was expressed at higher levels, suggesting that the protective role of this receptor is lost in tumor, regardless of metastasis (Figure 5b).

## 3. Discussion

Colorectal cancer is the most common malignant tumor characterized by inflammatory conditions promoted by immune cells and inflammatory mediators. Previously, we demonstrated the presence of an altered lipidomic profile, characterized by a high omega-6/omega-3 PUFAs ratio, in the red blood cell membranes of CRC patients, and this pro-inflammatory profile was also found in the tumor tissue of patients with synchronous metastases [8,12]. Starting from these findings, in this study we confirm the presence of an altered tissue fatty acids profile in CRC patients with synchronous metastasis, detecting high levels of the AA/EPA ratio, which is considered an adequate indicator of inflammation [13,16].

In this study, we used the lipidomic approach to detect the AA/EPA ratio levels in tumor tissue of subjects with metastases compared to those without metastases. Our findings not only confirm a pro-inflammatory condition closely associated with the tumor tissue, but also suggest a predictive role of AA/EPA ratio in the progression of CRC. 

COX-2 and 15-LOX-1, strongly linked to the oxidative metabolism of AA and EPA, exert opposite functions on pathogenesis of cancer in colon [19,24]. COX-2 is not expressed in the non-pathological colonic mucosa, while it is induced in the tumor microenvironment by pro-inflammatory stimuli such as bacterial lipopolysaccharides, IL-1β, IFN-γ and TNF-α [25]. Mice treated with prostaglandin E2, derived from COX-2, showed a drastic increase of intestinal tumor burden and a significantly higher incidence and multiplicity of colon cancer induced by AOM [26,27]. Selective COX-2 inhibitors, such as aspirin and other non-steroidal anti-inflammatory drugs (NSAIDs), are able to reduce prostaglandins levels leading to a reduction in tumor progression [28,29].

Conversely, 15-LOX-1 expression is down regulated in various human cancer, including CRC [19,30], and several evidences have demonstrated its anti-inflammatory role by activating PPAR-γ pathway [31,32]. A theoretical model has been proposed to explain the inverse association between 15-LOX-1 and COX-2 expression during colonic tumorigenesis [19]. According to this model, 15-LOX-1 inhibits nuclear factor-κβ (NF-κβ) and consequently COX-2 transcription [19]. The dual function, pro- and anti-inflammatory of COX-2 and 15-LOX-1, assures a balance within the cell between the eicosanoids produced. The disturbance of this equilibrium leads to a state of disorder and inflammation that could be implicated in colon cancer progression. 

Confirming these data, here we found high levels of COX-2 gene expression and a low gene expression of 15-LOX-1 in the tumor tissue of patients with metastases compared to those without metastases, justifying the enhanced levels of AA/EPA ratio detected in the same samples. 

Another possible mechanism linked to the inflammatory index AA/EPA ratio, could be the involvement of the endocannabinoid system (ECS) and the PPAR protein family. Several studies have demonstrated that an increase in endocannabinoid levels leads to activation of these systems [22,33,34]. The anti-inflammatory effects exerted by AEA and 2-AG are mediated by the induction of both CB2-R and PPAR-γ expression. Various attempts have been made to inactivate the enzymes that degrade endocannabinoids, in order to increase the levels of AEA and 2-AG in the tissue for their anti-tumor action [35,36]. COXs and LOXs play a role in controlling endocannabinoid levels, oxidizing AEA and 2-AG to subtract them from the system. In addition, low levels of PPAR-γ seem to lead to a consequent increase in COX-2 gene levels [37]. The reduction of gene and protein expression levels of both CB2-R and PPAR-γ found in the tumor tissues of patients with metastases compared to those without metastases, confirms that their negative regulation on cell inflammation and proliferation is lost in metastatic CRC.

This study wants to focus the attention on the use of lipidomics in the identification of metabolic profiles of complex diseases as CRC. Since the release of AA from membrane phospholipids to enter the cascade of prostaglandins synthesis and activation of cell response is a hallmark of cancer, lipidomics of inflammation allows the follow-up of the massive changes of lipid homeostasis happening in metastatic process. 

## 4. Materials and Methods 

### 4.1. Patients

Sixty-eight consecutive patients with histologically proven colorectal cancer were recruited by the Surgery Unit of the National Institute of Gastroenterology “S. de Bellis”, Castellana Grotte, Italy, from February 2017 to February 2019. 

Of these, 33 patients had synchronous metastases at the first diagnosis in the liver, in visceral lymph nodes, in bone and in lung. Before participation at the study, all subjects provided an informed consent form and the study was conducted in accordance with the Helsinki Declaration and approved by the Ethical Committee of IRCCS “S. de Bellis”, Castellana Grotte (Bari, Italy, number code: 32/CE/DE BELLIS, 27 October 2016). Samples of mucosa, take from macroscopically normal areas of intestine at 10 cm from neoplastic lesion, were stored at −80 °C until assayed. The clinical and histopathological features of all patients are shown in Table 1.

### 4.2. AA/EPA Ratio Assay

Tissue samples from CRC patients with and without metastases were used to evaluate the AA/EPA ratio. We used the extraction method of Folch with slight modifications [38,39]. Fatty acids extraction and preparation of fatty acid methyl esters (FAME) from tissue samples were carried out as previously described [12,40]. Briefly, about 20 mg of wet intestinal tissues were homogenized by adding 0.8 mL of ice cold 0.9% NaCl. 

Subsequently, were added 5.0 mL of chloroform:methanol (2:1, *v*/*v*) (Sigma-Aldrich, Milan, Italy) and the samples were mixed thoroughly and centrifuged at 1000× *g* for 10 min. Fatty acids containing in the lower layer, were put on in a new tube and dried by a centrifugal evaporator (Thermo Fisher Scientific, Waltham, MA, USA). The FAME were obtained by adding toluene and boron trifluoride-methanol solution 14% in methanol (Sigma-Aldrich, Milan, Italy) and incubating for 2 h at 80 °C. The samples were centrifuged after the addition of 5% aqueous sodium chloride solution and toluene, and the FAME obtained were injected into gas chromatograph (Thermo Fisher Scientific, Focus GC, Milan, Italy) equipped with auto-sampler, a split/splitless injector, Flame Ionization Detector (FID) and a hydrogen gas generator (Hy Gen 200, Claind Srl, Lenno, Italia). Separation of FAME was carried out on a capillary (SGE Europe Ltd., Milton Keynes, UK), as previously described [12]. Quantification of FAME, in particular AA and EPA methyl esters, was performed using a mixture of standards (Supelco 37-Component FAME Mix, Sigma-Aldrich, Milan, Italy).

### 4.3. Gene Expression Analysis

Total RNA was extracted from non-tumor adjacent mucosa and cancer samples using TRIzol™ Reagent (Invitrogen, US). cDNA was synthesized from 2 µg of total RNA using the iScript Advanced cDNA Synthesis Kit (Bio-Rad, Milan, Italy) following the manufacture’s instruction. To evaluated COX-2, 15-LOX-1, CB2-R, PPAR-γ gene expression levels has been used Real-time PCRs. In 20 µL of final volume were contained 2 µL of cDNA, a master mix with SYBR Green (iQ™ SYBR^®^ Green Supermix, Bio-Rad, Milan, Italy) and sense and antisense primers for the COX-2, 15-LOX-1, CB2-R, PPAR-γ and the β-actin gene (Table 2). β-actin was used as an internal loading control. Real-time PCRs were carried out in a CFX96 Real-Time PCR Detection System (Bio-Rad, Milan, Italy) using the following protocol: 45 cycles at 95 °C for 3 min, 95 °C for 10 s, 55 °C for 30 s followed by a melting curve to distinguish specific from non-specific products and primer dimers. Relative quantification was done using the ∆∆Ct method.

### 4.4. Western Blotting

Total protein extracts were obtained treating each tissue sample with total lysis buffer (Pierce Ripa buffer, Thermo Scientific, Rockford, IL, USA) supplemented with protease and phosphatase inhibitors (Thermo Scientific, Rockford, IL, USA). After homogenization and centrifugation, protein concentration was measured by a standard Bradford assay (Bio-Rad, Milan, Italy). Aliquots of 50 µg of total protein extracts from each sample were denaturated in Laemmli sample buffer and loaded into 4–12% pre-cast polyacrylamide gels (Bio-Rad, Milan, Italy) for western blot analysis. CB2-R (cat. n° ab 3561, Abcam, Cambridge, MA), PPAR-γ (cat. n° #2443, Cell Signaling Technology, Beverly, MA, USA), and β-actin (cat. n° #4970, Cell Signaling Technology, Beverly, MA, USA) were used as primary antibodies. After overnight incubation, the membranes were incubated with a horseradish peroxidase-conjugated secondary antibody (Bio-Rad, Milan, Italy). The proteins were detected by chemiluminescence (ECL, Thermo Scientific, Rockford, IL, USA) and each protein-related signal was obtained using the Molecular Imager Chemidoc^TM^ (Bio-Rad, Milan, Italy) and normalized against β-actin protein expression.

### 4.5. Statistical Analysis

Data were analyzed by the Student’s *t*-test or one-way analysis of variance (ANOVA) and Tukey’s multiple comparison test, where appropriate. The chi-squared test was used to investigate the association between tumor stage, histological tumor grade and AA/EPA ratio levels. Data are expressed as mean and Standard Deviation (DS), where appropriate. Differences were considered significant at *p* < 0.05. 

## 5. Conclusions

The high levels of AA/EPA ratio in metastatic patients with CRC induce an inflammatory microenvironment more susceptible to tumor progression. The importance of AA/EPA ratio is strictly connected with the need to identify new agents and new biomarkers to be introduced into clinical practice of CRC.

## Figures and Tables

**Table 1:** Descriptive table of the main clinical and histopathological characteristics of colorectal cancer patients with and without synchronous metastasis.

	*CRC Patients (n.68)*	
	No metastases (*n* = 35)	Metastases (*n* = 33)
Age	69.9 ± 13.8	68.7 ± 9.3
Sex		
Male	19	22
Female	16	11
Tumor Side		
Right (hepatic flexure, cecum and ascending colon)	17	12
Left (descending colon, sigmoid and rectum)	18	21
Tumor Stage (Clinical staging performed using UICC System)		
Stage I	5	2
Stage II	24	2
Stage III	5	19
Stage IV	1	10
Histological Grading		
Well-differentiated (G1)	4	2
Moderately differentiated (G2)	20	16
Poorly differentiated (G3)	11	15
Metastases Site		
Liver	0	13
Visceral lymph nodes	0	18
Bone	0	1
Lung metastases	0	1

**Table 2:** Sequences of amplification primers.

Gene	Primer
*COX-2*	
Forward	5′-TCTGGTCAATGGAAGCCTGT-3′
Reverse	5′-CAGCACTTCACGCATCAGTT-3′
*15-LOX-1*	
Forward	5′-CAGACGTGGCTGTGAAAGAC-3′
Reverse	5′-CAGGAAACCCTCGGTCCTG-3′
*CB2-R*	
Forward	5′-CCACAACACAACCCAAAGCCT-3′
Reverse	5′-ATCTCTGTCACCCAGCATTCC-3′
*PPAR-γ*	
Forward	5′-GGAAGACCACTCGCATTCCTT-3′
Reverse	5′-GTAATCAGCAACCATTGGGTCA-3′
*β-actin*	
Forward	5′-AAAGACCTGTACGCCAACACAGTGCTGTCTGG-3′
Reverse	5′-CGTCATACTCCTGCTTGCTGATCCACATCTGC-3′
